# Cytochrome *b*_5_: A versatile electron carrier and regulator for plant metabolism

**DOI:** 10.3389/fpls.2022.984174

**Published:** 2022-09-23

**Authors:** Chang-Jun Liu

**Affiliations:** Biology Department, Brookhaven National Laboratory, Upton, NY, United States

**Keywords:** cytochrome *b*_*5*_, cytochrome P450, lignin, flavonoids, unsaturated fatty acid, very long chain fatty acid, sugar transporter, ethylene signaling

## Abstract

Cytochrome *b*_5_ (CB5) is a small heme-binding protein, known as an electron donor delivering reducing power to the terminal enzymes involved in oxidative reactions. In plants, the CB5 protein family is substantially expanded both in its isoform numbers and cellular functions, compared to its yeast and mammalian counterparts. As an electron carrier, plant CB5 proteins function not only in fatty acid desaturation, hydroxylation and elongation, but also in the formation of specialized metabolites such as flavonoids, phenolic esters, and heteropolymer lignin. Furthermore, plant CB5s are found to interact with different non-catalytic proteins such as ethylene signaling regulator, cell death inhibitor, and sugar transporters, implicating their versatile regulatory roles in coordinating different metabolic and cellular processes, presumably in respect to the cellular redox status and/or carbon availability. Compared to the plentiful studies on biochemistry and cellular functions of mammalian CB5 proteins, the cellular and metabolic roles of plant CB5 proteins have received far less attention. This article summarizes the fragmentary information pertaining to the discovery of plant CB5 proteins, and discusses the conventional and peculiar functions that plant CB5s might play in different metabolic and cellular processes. Gaining comprehensive insight into the biological functions of CB5 proteins could offer effective biotechnological solutions to tailor plant chemodiversity and cellular responses to environment stimuli.

## Introduction

As sessile organisms, terrestrial plants have evolved remarkable metabolic capacity elaborating abundant primary metabolites to sustain their growth, development and reproductivity, and a vast variety of functionally specialized metabolites, such as fragrances, pigments, anti-fungal or anti-herbivory phytoalexins, lipidic surface polymers, and cell wall structural components, to cope with ever changing environmental challenges (Weng and Chapple, 2010). The fortification of land plant metabolic capacity is not only achieved by recruiting and evolving catalytic enzymes, but also by inheriting, co-evolving, and expanding many non-catalytic auxiliary proteins and cofactors in different metabolic processes.

**FIGURE 1:**
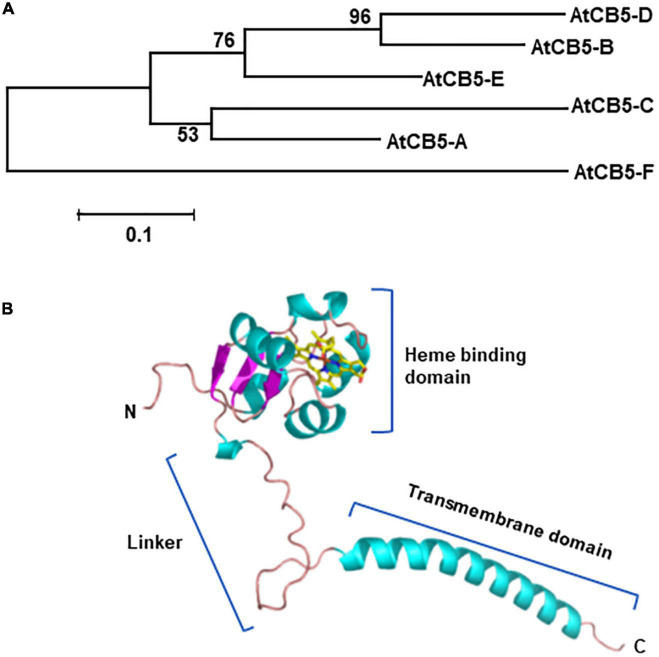
Phylogenetic relationship of Arabidopsis cytochrome *b*_5_ proteins **(A)** and structural model of one of the cytochrome *b*_*5*_ family member AtCB5-D **(B)**. Protein sequences of 6 Arabidopsis CB5 homologs were used in the analysis. The sequences were aligned with ClustalW integrated in the MEGAv.7.0 program. The evolutionary history was inferred using the neighbor joining method. The percentages (>50%) of replicate trees in which the associated genes clustered together in the bootstrap test (1,000 replicates) are shown next to the branches. The units represent the number of amino acid substitutions per site. Structural model was built with Alphafold 2. AtCB5-A: AT1G26340, cytochrome *b*_5_ isoform A. Also named as B5#6, CB5-A. AtCB5-B: AT2G32720, cytochrome *b*_*5*_ isoform B. Also named as B5#4, CB5-B, CYTB5-D. AtCB5**-C**: AT2G46650, cytochrome b_5_ isoform C. Also named as B5#1, CB5-C, CYTB5-C. AtCB5-D: AT5G48810, cytochrome *b*_*5*_ isoform D. Also named as B5#3, CB5-D, ATB5-B, CYTB5-B. AtCB5-E: At5G53560, cytochrome *b*_*5*_ isoform E. Also named as B5#2, CB5-E, ATB5-A. AtCB5-F: AT1G60660, cytochrome *b*_*5*_ like protein. Also named as ATCB5LP or CB5LP.

Cytochrome *b*_5_ (CB5) is a small heme-binding protein found in all life kingdom, including bacteria, fungi, mammals/human, and plants. Typically CB5 protein is a tail-anchored membrane protein and possesses a single transmembrane domain and a tail region near its C-terminus to regulate intracellular localization to the endoplasmic reticulum (ER) and/or the outer mitochondrial membranes (Schenkman and Jansson, 2003). The key characteristic of CB5 is the possession of a highly conserved heme-binding motif (-HPGG-) that locates in the large N-terminal domain typically exposed to the cytosol (Schenkman and Jansson, 2003) (Figure 1). With bound heme molecule, CB5 possesses redox potential of ∼20 mV, capable of accepting and transferring a single electron. It can be reduced either by NADH-dependent cytochrome *b*_5_ reductase (CBR) or by NADPH-dependent cytochrome P450 reductase (CPR), therefore, shuttling electron(s) in either NADH-CBR-CB5 chain or NADHP-CPR-CB5 pathway at the ER membrane to the terminal acceptors (proteins or enzymes) involved in oxidation/hydroxylation reactions (Vergeres and Waskell, 1995; Porter, 2002; Schenkman and Jansson, 2003). As an electron donor protein, CB5 has been extensively studied in mammals in respect to its roles in cellular detoxification and drug metabolism. It has been found to function in anabolic metabolism of fatty acids and steroids, catabolism of xenobiotics and compounds of endogenous metabolism (Vergeres and Waskell, 1995; Porter, 2002; Schenkman and Jansson, 2003).

In contrast to the single copy of *CB5* gene in mammals and yeast (Porter, 2002; Kandel and Lampe, 2014), higher plants including Arabidopsis have evolved multiple copies of the gene (Hwang et al., 2004; Maggio et al., 2007). The significant expansion of the CB5 family in the plant kingdom implicates that this group of heme-containing proteins might play broader and more complicated cellular and metabolic functions than their mammalian/human counterparts. Similar to animal/human ones, the ER-resident CB5s in plants, together with their redox partners, have also been implicated as the electron carriers to deliver reducing equivalents from pyridine nucleotide cofactor to the processes of acyl-CoA/fatty acid desaturation, hydroxylation, and triple bond formation (Smith et al., 1992; Napier et al., 1997; Lee et al., 1998; Broadwater et al., 2002; Kumar et al., 2012), sphingolipid long-chain base hydroxylation and desaturation (Napier et al., 2003; Nagano et al., 2012), or sterol desaturation (Rahier et al., 1997). Moreover, sporadic evidence indicate that plant CB5 proteins also function in the redox reactions of broader metabolic processes, in particular, plant specialized metabolism for synthesis of flower pigment anthocyanins, UV-resistance phenolic esters, pathogen defensing phytoalexins, and the cell wall structural component lignin. Furthermore, either as the redox protein or structural component, plant CB5s likely serve as metabolic or signaling regulators, connecting hormone or sugar signals, or environmental stimulus with particular metabolic or cellular processes. This article overviews the studies pertaining to plant CB5s and discusses their biological functions in different metabolic and physiological processes, with which we expect to trigger more research interests in deciphering the metabolic and cellular roles of this group of non-catalytic proteins in plant growth, development and defense responses.

## Evolutionary expansion of plant cytochrome *b*_5_ family

Cytochrome *b*_5_ are found in animals/human, plants, yeasts and purple phototrophic bacteria, indicative of their early emergence and evolution (Schenkman and Jansson, 2003). Yeast and human genomes contain a single copy of *CB5* gene, although the human gene generates two isoforms via alternative mRNA splicing. The isoform 1 locates to the cytoplasmic side of the ER, while isoform 2 is in cytoplasm (Porter, 2002; Schenkman and Jansson, 2003; Kandel and Lampe, 2014). By contrast, the genomes of higher plants typically evolve multiple *CB5* genes (Smith et al., 1992; Napier et al., 1995; Fukuchi-Mizutani et al., 1999; Hwang et al., 2004; Kumar et al., 2006; Maggio et al., 2007; Kumar et al., 2012). *Arabidopsis thaliana* possesses five canonical *CB5* genes encoding isoforms AtCB5-A (At1g26340), AtCB5-B (At2g32720), AtCB5-C (At2g46650), AtCB5-D (At5g48810), and AtCB5-E (At5g53560), which share amino acid identities ranging from 40 to 70% (Figure 1A). All five isoforms possess the conserved features of CB5 family: A N-terminal water-soluble/cytosolic heme binding domain, a highly flexible linker region that possibly provides the directional freedom required for an efficient complex formation with terminal enzymes such as P450s, and a C-terminal transmembrane domain that anchors protein to either the ER or mitochondria/chloroplast, followed by a short luminal tail (Ahuja et al., 2013; Pandey et al., 2013) (Figure 1B). In addition to the five canonical CB5s, a heme-binding protein encoded by At1g60660 possesses a short transmembrane domain at its N-terminus, which is defined as CB5-like protein, AtCB5F or AtCB5LP (Hwang et al., 2004; Maggio et al., 2007) (Figure 1A); among the annotated five canonical CB5 proteins, AtCB5-B, -C, -D and -E share more sequence similarity; each of their tail sequences carry a conserved ER-targeting motif (-R/H-x-Y/F-) and are proven or predicted to localize to the ER membrane (Hwang et al., 2004; Maggio et al., 2007), while AtCB5-A is demonstrated to localize to the chloroplast envelope (Maggio et al., 2007). Similarly, rice genome encodes eight CB5 isoforms and soybean encompasses about 11 putative CB5 members (Kumar et al., 2006). Overall, the number of plant CB5 family members is much larger than that in yeasts and animals. The exact evolutionary significance of such large expansion of plant CB5 family remains to be determined. Given the fact that sessile plants need to produce a plethora of defense-related specialized metabolites to deal with ever changing environment, CB5 family might co-evolve with the massive expansion of plant metabolic enzymes such as cytochrome P450 superfamily enzymes to invent metabolic complexity, versatility and robustness.

It is worthwhile to note that the nomenclature of plant CB5 proteins is inconsistent and somehow confusing in the literatures. For example, on one hand, the same Arabidopsis CB5 member has a few different names; on the other hand, a same given name denotes to the different family members in different studies (see Figure 1 Legend for more information). Attention should be paid to avoid potential misunderstanding.

## Cytochrome *b*_5_ functions as redox cofactor in biosynthesis of fatty acids

### Fatty acid desaturation and hydroxylation

Analogous to animal/human counterparts, plant CB5s are widely implicated as a redox cofactor in the biosynthesis of plant specialty fatty acids, and the membrane or cuticular lipids. CB5s are commonly believed to associate with NADH-dependent CBR, and shuttle reducing equivalents from reductant NADH to the terminal acceptors. In most cases, CB5 transfers electrons to the ER-localized non-heme, iron-containing enzymes involved in fatty acid desaturation, hydroxylation, and elongation (Napier et al., 2003; Nagano et al., 2012). Hydroxyl fatty acids such as ricinoleic acid (12-hydroxyoctadec-*cis*-9-enoic acid) are the important feedstocks for industrial applications; while polyunsaturated fatty acids such as ω-3 fatty acids are the essential nutrient components beneficial to human health. Early biochemical studies discovered that CB5 proteins presented in many oilseed species. The CB5 protein in microsomal preparation is necessarily required for the conversion of oleate to polyunsaturated linoleate in safflower (*Carthamus tinctorius*) (Smith et al., 1990), or to ricinoleic acid in castor bean (Smith et al., 1992). Applying anti-CB5 antibody raised against the hydrophilic CB5 fragment from cauliflower (*Brassica oleracea*) to the microsomal enzyme assay system inhibits both Δ12-hydroxylase and Δ12-desaturase activities in the prepared microsomes from castor bean, suggesting that CB5 is the indispensable redox component for the membrane resident, non-heme fatty acid desaturase and hydroxylase systems (Smith et al., 1992). Arabidopsis CB5 is reduced by either NADH-dependent CBR or NADPH-dependent CPR (Fukuchi-Mizutani et al., 1999). Disruption of Arabidopsis *CBR* in either the wild type background or the transgenic line with overexpression of castor fatty acid 12-hydroxylase (FAH12) significantly depleted the accumulation of 18 carbon hydroxy fatty acids and unsaturated fatty acids in developing seeds, suggesting that the conventional NADH-CBR-CB5 electron transfer chain is responsible for fatty acid hydroxylation and desaturation (Kumar et al., 2006).

To discriminate the potential differential contributions of CB5 isoforms in fatty acid synthesis, Kumar et al. (2012) co-expressed Arabidopsis ER-resident CB5s, AtCB5-B, -C, -D and -E with Arabidopsis fatty acid desaturase FAD2 and FAD3, respectively, in a yeast *cb5* mutant to measure the production of ω-3 and ω-6 desaturation of C16 and C18 fatty acids. All four CB5s were able to enhance the accumulation of either di- or tri-unsaturated fatty acids, compared to the yeast cells expressing FAD2 or FAD3 alone. However, they exhibited distinct effects on the production of unsaturated fatty acid species. AtCB5-C and -D (denoted as Cb5-C and Cb5-B in the study) significantly enhanced the yield of 16:2 and 18:2 di-unsaturated fatty acids, about 1.5–2-fold higher than did AtCB5-B (Cb5-E) or AtCB5-E (Cb5-A) with FAD2; whereas AtCB5-B and AtCB5-E, when co-expressed with FAD3, yielded the better production of 18:3 tri-unsaturated fatty acids (Kumar et al., 2012). Although the data from yeast heterologous system might not necessarily reflect the *in planta* functionalities of CB5s, the study implicates that Arabidopsis CB5 isoforms exhibit differential stimulatory effects on FAD2 and FAD3 activities. Further genetic evidence is required for validating the functional differentiation of CB5s in the production of unsaturated fatty acid *in planta*.

### Very long chain fatty acid elongation

Very long chain fatty acids (VLCFAs) are the compounds with an acyl chain of 18 carbons and longer. In plants, VLCFAs are incorporated into various lipid pools, including the neutral seed storage lipids triacylglycerols, the membrane constituents and signaling molecules phospholipids and sphingolipids, and the extracellular epicuticular waxes and suberin (Bach and Faure, 2010; Haslam and Kunst, 2013). The content of VLCFAs increases in response to various environmental stresses such as salt, wounding, drought, hypoxia, and pathogen infection (De Bigault Du Granrut and Cacas, 2016). VLCFAs are synthesized via sequential addition of two carbons to the C16 or C18 acyl-CoAs, catalyzed by elongase complex in the ER. This complex is composed of four enzymes, 3-ketoacyl coenzyme A synthase (KCS), 3-ketoacyl-CoA reductase (KCR), 3-hydroxyacyl-CoA dehydratase (HCD) and *trans*-2,3-enoyl-CoA reductase (ECR) that carry on sequential reactions of acyl-CoA condensation, reduction, dehydration then reduction (Haslam and Kunst, 2013). In Arabidopsis, the CoA condensing enzymes are encoded by 21 *FAE1*-like *KCS* genes and 4 *ELO*-like genes (*AtELO1, At3g06460; AtELO2, At3g06470; AtELO3, At1g75000; and AtELO4, At4g36830*) (Dunn et al., 2004; Joubes et al., 2008). All four ELO homologs localize to the ER-membrane but only AtELO1 and AtELO2 contain a HXXHH motif that is reminiscent of the His-rich motif generally conserved in the integral membrane-bound desaturases, hydroxylases, and elongases (Fox et al., 1993; Mitchell and Martin, 1997; Oh et al., 1997). The histidine residues of this motif coordinate a μ-oxo diiron cluster (Fe–O–Fe) that forms part of the catalytic core. AtELO3 and AtELO4 possess neither the characteristic HXXHH motif nor the conserved amino acids, but remain their condensation activities (Quist et al., 2009; Nagano et al., 2019). Interestingly, split ubiquitin membrane yeast-two-hybrid (Y2H) and biomolecular fluorescence complementation (BiFC) assay revealed that AtCB5-B interacts with AtELO1 and AtELO2 but not AtELO3 andAtELO4 in yeasts and in plants (Quist et al., 2009; Nagano et al., 2019), suggesting that the His-rich motif might be a critical structural feature for the physical interaction between those non-heme enzymes and the CB5 proteins but such interaction might not be critical for the condensation activity. This invokes an interesting question why AtELO1 and AtELO2 need to interact CB5 protein. BiFC assay also revealed that both AtCB5-B and AtELO2, respectively, interact with or are in close proximity to the VLCFA elongase complex enzymes KCR1, PAS2/HCD, and CER10/ECR (Nagano et al., 2019), implicating that AtCB5-B and elongase components might form a large protein complex *in planta*. Within the elongase complex, KCR1 and CER10/ECR catalyze acyl-CoA reductions. Their reactions require reducing power. Therefore, CB5 integrated into the VLCFA elongase complex might serve as a necessary electron donor for the reductase activity. On the other hand, electrons are not needed for the ELO/KCS-catalyzed condensation or PAS2/HCD-catalyzed dehydration. The direct physical interactions between those proteins/enzymes and AtCB5 infer that CB5 may potentially play the roles beyond as an electron donor, for instance, as allosteric stimulator for the elongase components.

### Very long chain alkane synthesis

The elongated acyl-CoAs can serve as metabolic precursors for the formation of several classes of lipids. Two distinct biosynthetic pathways direct them to the waxes: The decarbonylation pathway and the reduction pathway. Decarbonylation process begins with the production of aldehydes from acyl-CoA by acyl-CoA reductase, followed by a decarbonylation catalyzed by an aldehyde decarbonylase to produce alkanes with odd-chains. The resulting alkanes are then hydroxylated to the secondary alcohols, which are finally oxidized to ketones (Bernard and Joubes, 2013). In Arabidopsis, the wax-associated protein ECERIFERUM1 (CER1) and ECERIFERUM3 (CER3) are the core components of very long chain alkane synthesis complex, and two proteins associate together (Bourdenx et al., 2011; Bernard et al., 2012). Both split ubiquitin Y2H and split luciferase assays revealed that CER1 and a CER1-like protein physically interact with all four ER-localized CB5 proteins (AtCB5-B, -C, -D and -E) (Bernard et al., 2012; Pascal et al., 2019). Co-expression of AtCB5-B with CER1/CER3 complex significantly increased the production of alkanes with distinct chain-length specificity in yeast. Both CER1 and CER3 possess tripartite His-rich motifs; point mutations of the His-rich motifs in CER1 diminished the alkane-forming activity of the CER1/CER3/AtCB5-B complex, but mutations of the His-rich motifs in CER3 did not compromise alkane production (Bernard et al., 2012; Pascal et al., 2019). The data suggest that AtCB5 protein likely serve as the electron donor mediating electron transfer to the catalytic site of the CER1 or CER1-like enzyme, which is necessary for the product specificity and overall productivity of very long chain alkane synthesis.

### Sphingolipid fatty acid hydroxylation

Sphingolipids are the important structural components of plasma membrane (PM), accounting for more than 40% of its lipids. Sphingolipids are also found in the Golgi network and in endosomes (Bach and Faure, 2010; Haslam and Feussner, 2022). Complex sphingolipids are composed of various head groups and a ceramide formed by linking long chain base amine group to fatty acids. A major structural feature of plant sphingolipids is the hydroxylation of the C-2 position in the fatty acid molecules. More than 90% of the sphingolipids in Arabidopsis contain 2-hydroxy fatty acids. Sphingolipids are also composed of very-long-chain fatty acids (VLCFAs), which have more than 20 carbons (Haslam and Kunst, 2013; Haslam and Feussner, 2022). Similar to other fatty acid hydroxylases, e.g., yeast homolog ScFAH1, Arabidopsis sphingolipid fatty acid 2-hydroxylases AtFAH1 and AtFAH2 contain five conserved His-rich motifs [HX2-3(XH)H] that form part of di-iron cluster to receive electrons. Like CER1, both AtFAH1 and AtFAH2 physically interact with all five Arabidopsis CB5 proteins (Nagano et al., 2009), suggesting CB5 might be the indispensable electron donor in FAH-catalyzed sphingolipid 2-hydroxylation.

### Connector of environmental signals and fatty acid biosynthesis

Interestingly, CB5 proteins not only interact with electron acceptors involved in fatty acid desaturation, hydroxylation and elongation, but also physically associate with the non-catalytic regulatory component relevant to the lipid biosynthesis. In Arabidopsis, the cell death suppressor Bax inhibitor-1 (BI-1), an ER membrane protein, functionally associates with various environmental stresses. Overexpression of BI-1 suppressed cell death and conferred tolerance to the oxidative, salinity and drought stresses in Arabidopsis, tobacco, rice, and sugarcane (Isbat et al., 2009; Ishikawa et al., 2010; Ishikawa et al., 2011; Ramiro et al., 2016). Moreover, overexpression of BI-1 also increased the production of the 2-hydroxylated VLCFAs in the normal condition and promoted the rapid synthesis of 2-hydroxy VLCFAs in the plants under oxidative stress, suggesting BI-1 is responsive to environmental stimuli and regulates fatty acid 2-hydroxyalse activity (Nagano et al., 2009). Notably, AtBI-1 physically interacts with the ER-resident CB5s and the yeast ScFAH1 that contains a CB5-like domain, but not with AtFAHs that lack such domain. Nevertheless, CB5 directly interacts with AtFAHs (Nagano et al., 2009). Therefore, CB5 likely acts as a linker mediating the functional association of AtBI-1 with AtFAHs. Furthermore, co-immunoprecipitation using AtBI-1 as bait pulled down VLCFA synthesizing enzymes, including AtELO2, KCS10, KCR, PAS2, CER10, and AtCB5, suggesting CB5 may also mediate the association of AtBI-1 with VLCFA elongase complex (Nagano et al., 2019). Since AtBI-1 interacts with Arabidopsis calmodulin (AtCaM) to perceive environmental simulation (Ihara-Ohori et al., 2007), it is possible that AtBI-1 transduces environmental signals from AtCaM through interacting with CB5 to regulate CB5-FAH complex activity thus enhancing 2-hydroxylation of fatty acids, and/or modulating CB5-elongase complex function. Consequently it might alter sphingolipid structures and properties thus inactivating the ceramide-mediated signal transduction in cell death or reinforcing membrane microdomain, which ultimately suppresses plant cell death process. In such processes, CB5 not only acts as an electron carrier but also as a regulator conveying environmental signals from AtBI-1.

## Cytochrome *b*_5_ acts as electron donor for cytochrome P450-catalyzed reactions

When pioneer land plants migrated from aquatic habitats to terrestrial environment ∼500 million years ago, the overwhelming biotic and abiotic environmental stresses become the primary driving force to accelerate the evolution of plant metabolic capacity. The most outstanding evolution event is the massive expansion of cytochrome P450 systems, which eventually leads to by far the largest family of enzymes in plant metabolism (e.g., 245 super family members emerging in Arabidopsis) (Bak et al., 2011; Nelson and Werck-Reichhart, 2011). The cytochrome P450 enzymes have a key function for generating the chemical diversity, which is the hallmark of plants compared with animals. Plant P450s are crucial for the biosynthesis and metabolism of fatty acids, phytosterols, plant growth regulators, and a variety of plant specialized metabolites, including phenylpropanoids, terpenoids, glucosinolates, and indoalkaloid phytoalexins (Mizutani and Ohta, 2010; Bak et al., 2011). Interestingly, although P450 enzymes play central roles in various metabolic processes, this family of heme-containing oxidases are functionally self-insufficient. Like non-heme-containing desaturases and hydroxylases in fatty acid modification, eukaryotic microsomal P450s require redox partner(s) to deliver two electrons to its catalytic center for cleavage of oxygen molecule in each catalytic cycle (Hannemann et al., 2007). Typically, microsomal P450s rely on diflavin reductase, the cytochrome P450 oxidoredcutase (CPR), to transfer electrons from cofactor NADPH to the prosthetic heme group of P450 (Urban et al., 1997; Jensen and Moller, 2010). Evidence from mammalian P450 systems also reveal that CB5 can be reduced by NADPH-dependent CPR (Enoch and Strittmatter, 1979; Vergeres et al., 1995; Porter, 2002). In such case, CB5 delivers second electron to the P450 ferrous-O_2_ complex (Durr et al., 2007). CB5 augments a subset of P450-catalyzed reactions in mammalian systems. It displays either stimulation, no effect, or inhibition effects in different cases (Porter, 2002; Bart and Scott, 2017). In plants CB5s are also indispensable for some of P450-catalzyed reactions in the synthesis of specialized metabolites or cell wall structural component lignin. In petunia, disruption of a *cb5* locus, *DifF*, resulted in the discoloration of its flower petals, which coincides with the compromised activity of flavonoid 3′, 5′-hydroxylase (F3′5′H), the P450 enzyme leading to the synthesis of flavonols and the core structure of anthocyanidin delphinidin (de Vetten et al., 1999). Furthermore, a Arabidopsis CB5 family member, the AtCB5-D, was recently discovered as the key determinant for the synthesis of syringyl lignin subunits and the related soluble 5-hydroxylated phenolics, sinapoyl esters (Gou et al., 2019). Disruption of *AtCB5-D* resulted in more than 60% reduction of S-lignin deposition without impairment of guaiacyl lignin accumulation in the cell walls of Arabidopsis stem and ∼70% reduction of the wild type accumulation level of sinapoyl malate, a photoprotectant phenolic ester accumulated in the leaf epidermis of *Brassicaceae* family (Shirley et al., 2001; Milkowski and Strack, 2010). The loss of AtCB5-D primarily impaired the activity of ferulate 5-hydroxyalse 1 (AtF5H1, CYP84A1) that is a key branch point enzyme catalyzing benzene ring 5-hydroxyaltion and leading to the S-lignin monomer formation in angiosperms. The loss of the function of *AtCB5-D* also significantly suppressed the accumulation of α-pyrones, the compounds yielded from the activity of AtF5H2 (CYP84A4, At5G04330), a close paralog of AtF5H1 in Arabidopsis (Weng et al., 2012). In *cb5d* stem, the contents of the major α-pyrones arabidopyl alcohol and iso-arabidopic acid were reduced by ∼80% compared to the WT (Gou et al., 2019). However, disruption of *AtCB5-D* did not affect the accumulation of seed flavonol quercetin, the metabolite resulted from flavonoid 3′-hydroxylase (F3′H, CYP75A1) activity (Gou et al., 2019). AtF3′H is an evolutionarily close homolog of AtF5H1 and AtF5H2 (Weng et al., 2008). These data suggest that AtCB5-D possesses relatively strict specificity and supports a particular set of P450 enzymes in planta. Interestingly, both Y2H and BiFC assays revealed that AtCB5-D physically interacts with all three monolignol biosynthetic P450s, cinnamate 4-hydroxyalse (C4H), *p*-coumaryl ester 3′-hydroxyalse (C3′H) and F5H1, but genetic data clearly showed that AtCB5-D functionally only associates with F5H specific for the synthesis of S-lignin monomer and the related 5-hydroxylated phenolics (Gou et al., 2019). Therefore, it is of high interest to explore what factor(s) determine CB5’s functional speciation with P450 enzymes. On the other hand, AtCB5-D shares high sequence identity with other ER-localized CB5 members (44∼68%), particularly with AtCB5-B (68%) at amino acid level (Figure 1A). Although those ER-resident CB5 members also appear to interact/associate with monolignol biosynthetic P450s in Y2H or BiFC assay, only AtCB5-D imposes effects on lignin biosynthesis. It is intriguing to determine how CB5s distinguish their functionalities at the structural and/or molecular levels.

Although lack of the in-depth characterization, AtCB5-C was casually linked to the glucosinolate biosynthesis in a condition-specific manner (Vik et al., 2016). Glucosinolates, also known as mustard oil glucoside, are nitrogen- and sulfur-containing small molecule bioactive compounds found in the order *Brassicales*, including Arabidopsis and oilseed rape (*Brassica napus*). They function as defense compounds against insects and pathogen infection (Halkier and Gershenzon, 2006) but are also of great interest to humans due to the flavors they impart to various condiments and for their potential anticancer effects (Traka and Mithen, 2009). The function of AtCB5-C does not seem to be essential for the overall glucosinolate biosynthesis but it influences accumulation of particular long chain aliphatic glucosinolate species, especially when induced by MeJA treatment (Vik et al., 2016), which infers the potential functional association of this CB5 protein with CYP79F enzyme that catalyzes the key step in the biosynthesis of long chain aliphatic glucosinolates.

As electron shuttle intermediate, CB5s can transfer two electrons from NADH through CBR to the terminal enzymes, independent of NADPH-CPR; or transfer the second electron to oxyferrous P450 from CPR (Porter, 2002; Schenkman and Jansson, 2003). Arabidopsis CB5s are reduced both by CBR and CPR. However, both reductases display strict specificity toward pyridine nucleotide cofactors NADH and NADPH in reducing CB5 proteins. The recombinant AtCBR specifically utilizes NADH to reduce AtCB5; whereas AtCPR shows a sharp specificity to NADPH (Fukuchi-Mizutani et al., 1999). Notably, disrupting the ER-localized CBR in Arabidopsis resulted in no significant impairment on mature stem lignin biosynthesis, although a slight (but not statistically significant) reduction of S-lignin level occurred in the knock-out mutant (Gou et al., 2019). By contrast, disrupting Arabidopsis *CPR2*, namely *ATR2*, suppressed both G- and S-lignin synthesis up to 50% (Sundin et al., 2014; Gou et al., 2019). These data implicate that AtCB5-D in stem primarily couples with NADPH-CPR electron transport pathway for lignin synthesis. Collectively, CB5s might act as versatile electron carriers *in planta* that associate with different electron transfer chains for synthesis of distinct classes of metabolites.

## Cytochrome *b*_5_ functions more than as an electron carrier

### Sugar sensing through interactions with sugar transporters

While CB5 is commonly documented as an electron shuttle intermediate, sporadic evidence also suggest some non-conventional roles it fulfils. An early study revealed that human/mammalian CB5 with its heme molecule replaced with manganese-protoporphyrin IX was unable to accept and transfer electrons from CPR or CBR but retained the ability in decreasing the *Km* values of P450 enzymes when it was included with the reconstituted P450 systems containing CYP2B4 or CYP1A2 (Morgan and Coon, 1984). Moreover, the apo form CB5 devoid of heme is still able to enhance CYP3A4-catalyzed reactions as efficiently as does the holoprotein, suggesting that CB5 might act as an allosteric stimulator to tune P450 activity (Porter, 2002). While it remains to be determined whether plant CB5s also possess allosteric stimulation effect on the oxidase-catalyzed reactions, an apple (*Malus domestica*) ER-resident CB5 protein, MdCYB5, was found to physically interact with the plasma membrane (PM) – localized, low-affinity sucrose transporter MdSUT1, and sorbitol transporter MdSOT6. The interactions tune the affinities of both sugar transporters, allowing plant cells to adapt to sugar starvation (Fan et al., 2009). MdCYB5 is the close homolog of AtCB5-E (designated as AtCYB5-2/A in the study). The pair-wise interactions of MdCYB5 with MdSUT1 and MdSOT6 were well validated through split ubiquitin Y2H assay, co-immunoprecipitation/pull down, and *in planta* BiFC assay. In the yeast system, the low sugar supply enhances the interaction of MdCYB5 with either sugar transporters. Co-expression of MdCYB5 with MdSUT1 or MdSOT6 promotes the uptake of sugars and reduces the *Km* value of either transporters toward their substrate sucrose or sorbitol, thus increasing transporter affinity to sugar (Fan et al., 2009). Hypothetically MdCYB5 with its C-terminal tail anchored to the ER membrane interacts with the PM-localized sugar transporter MdSUT1 or MdSOT6, thus forming an MdSUT1-MdCYB5 or MdSOT6-MdCYB5 bimolecular complex in response to the low sucrose or sorbitol supply. The interaction enhances the affinity of MdSUT1 or MdSOT6 to its substrate sugar, thereby stimulating sugar uptake to maintain a relatively stable sugar level inside of the cell; whereas, under high sugar supply condition, the transporter-MdCYB5 interactions are attenuated and the affinity of the transporters to sugar substrate returns to the normal level (Fan et al., 2009). Besides apple CB5, the Arabidopsis CB5s are also functionally associated with sucrose transporter AtSUT4, the homolog of MdSUT1 but localized to tonoplast membrane instead of PM. All five Arabidopsis canonical CB5s physically interact with AtSUT4 (Li et al., 2012). Similar to the observed interaction of MdCYB5 and MdSUT1, sucrose but not glucose represses the interaction between AtSUT4 and AtCB5-E (Li et al., 2012). Despite the lack of adequate genetic validation, these several lines of biochemical evidence implicate that CB5 proteins might allosterically modulate sugar transporter activity in response to sugar/carbon source availability. The interaction of CB5 with sugar transporter tunes the transport kinetics, thereby sustaining cellular sugar/carbon homeostasis or modulating sugar signaling.

### Linking to ethylene signaling

Ethylene plays important roles in plant growth, development, and stress responses. It is often considered as an “aging” hormone due to its role in accelerating such developmental processes as ripening, senescence, and abscission. Ethylene is perceived by a family of receptor proteins that repress ethylene responses when ethylene is absent. Repression function of the ethylene receptor ETR1 depends on an integral membrane protein, REVERSION TO ETHYLENE SENSITIVITY1 (RTE1). The RTE1 protein acts at the upstream of ETR1 in the ER membrane and Golgi apparatus and serves as a molecular chaperone stabilizing or promoting the active signaling conformation of ETR1 (Dong et al., 2008; Resnick et al., 2008). Y2H and BiFC assays reveal that four Arabidopsis ER-localized CB5 isoforms (AtCB5-B, -C, -D, and -E) interact with RTE1 (Chang et al., 2014). Consistent with their interactions, *atcb5* mutants phenocopy Arabidopsis *rte1* line, showing partial suppression of *etr1-2* ethylene insensitivity. The AtCB5s exhibit partial functional redundancy. The single mutants of *atcb5-b*, *-c* and *-d* appear similarly to the wild type, but their double mutants display slight ethylene hypersensitivity. Conversely, over-expression of *AtCB5-D* confers a reduced ethylene sensitivity similar to that did the *RTE1* overexpression. These findings suggest an unexpected regulatory role of CB5 protein, i.e., *via* partnering with RTE1 to promote ETR1-mediated repression of ethylene signaling. In this process, AtCB5 may activate RTE1 through redox modification, thus linking cellular redox status with ethylene signaling (Chang et al., 2014). Since AtCB5-D is also an indispensable electron carrier involved in S-lignin biosynthesis, it therefore may serve as a regulatory/metabolic hub coordinating ethylene signaling and lignin synthesis in response to the cellular redox status. It is known that coordinated ethylene and auxin signaling plays an essential role in initiating and programming organ abscission. In the abscission zone, lignin is synthesized in secession cells to restrict cell wall processing enzymes to the precise area thus controlling precision process of cell walls for organ separation (Lee et al., 2018). The functional association of AtCB5-D with both ethylene signaling and lignin biosynthesis implicates that this redox protein might play exquisite regulatory roles coordinating cellular and biochemical activities in leaf, flower, or fruit abscission process.

## Biotechnological applications

The collective information suggests that plant CB5s exhibit versatile cellular functions and are involved in more complicated metabolic and signaling processes relative to their yeast, and animal counterparts. This family of proteins not only serve as conventional electron donor to drive redox reactions but may also act as physiological regulator functioning in environmental stress response, sugar and hormonal signaling processes. The electron donor function of CB5s in the defined metabolisms makes them an essential target in metabolic engineering for enhancing the production of high value bioproducts. In an effort to produce artemisinic acid, the precursor of anti-malarial drug artemisinin, in heterologous yeast (*Saccharomyces cerevisiae)* system, expression of *Artimisia annua* P450 enzyme CYP71AV1 with its cognate reductase (*CPR1*) accelerated amorphadiene oxidation, producing artemisinic acid at the yield of 115 mg/L (Ro et al., 2006). However, the engineered yeast cells suffered severe oxidative stress due to the poor coupling between cytochrome P450 and its reductase thus releasing ROS (Paddon and Keasling, 2014). Integration of a *A. annua* CB5, as well as artemisinic aldehyde dehydrogenase (*ALDH1*) and NAD-dependent artemisinic alcohol dehydrogenase (*ADH1*), together with CYP71AV1 and CPR1 significantly improved artemisinic acid yield (up to 25 g/L) (Paddon et al., 2013). Similarly, when using the engineered yeast cells to produce glycyrrhetinic acid (GA), the most essential ingredient in licorice with outstanding anti-inflammatory activity and the wide usage in medicine and cosmetics industries, introduction of the entire heterogeneous biosynthetic pathway of GA, including CYP88D6 and CYP72A154 combined with β-amyrin synthase and a *Arabidopsis thaliana* CPR into *S. cerevisiae*, only produced of 2.5 mg/L of β-amyrin and 14 μg/L of GA. However, further incorporation of a CB*5* from *Glycyrrhiza uralensis* resulted in eightfold enhancement of GA production. Furthermore, combining GuCB5 with other MVA pathway genes from *S. cerevisiae*, GA concentration was improved by 40-folds during batch fermentation (Wang et al., 2019). Both cases exemplify the significance of application of redox component CB5 in metabolic engineering to enhance the production of the desired metabolites.

## Conclusion and perspectives

**FIGURE 2:**
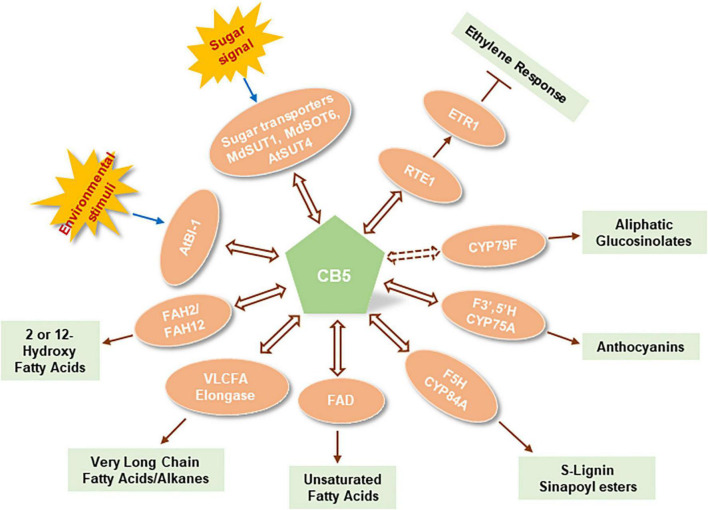
Functional association of plant cytochrome *b*_*5*_s with biological factors in different metabolic and cellular processes. AtBI-1, *Arabidopsis thaliana* Bax inhibitor-1; AtSUT4, *Arabidopsis thaliana* sucrose transporter 4; MdSUTTI, *Malus domestica* sucrose transporter 1; MdSOT6, *Malus domestica* sorbitol transporter 6; *RTE1*, Reversion to ethylene sensitivity 1; *ETR1*, Ethylene receptor 1; CYP79F, Cytochrome P450 superfamily enzyme member 79F; F3′, 5′H, Flavonoid 3′, 5′-hydroxylase; F5H, Ferulate 5-hydroxylase; FAD, Fatty acid desaturase; VLCFA Elongase, Very long chain fatty acid/alkane elongase enzyme complex; FAH2, Fatty acid 2-hydroxylase; FAH12, Fatty acid 12-hydroxylase. Double head arrows with solid line indicates the experimentally recognized interactions; Double head arrow with dashed line indicates the putative functional association.

Cytochrome *b*_*5*_ is a well-known electron shuttle intermediate in yeasts, and mammals/humans. It is involved in different oxidation/reduction reactions for biosynthesis of endogenous compounds such as steroids, vitamins, and fatty acids, and for the metabolism of xenobiotics and drugs (Schenkman and Jansson, 2003). In the endomembrane P450 system, mammal/human CB5 modulates P450 catalysis with either stimulating or inhibiting effects. Functionally interacting with P450s, mammal/human CB5 either plays a pure redox role of electron delivery or acts as an allosteric modulator of P450 conformation (Porter, 2002). CB5 family is largely expanded in plants, which strongly implicates its more complicated and diverse cellular/biological functions. Nevertheless, in contrast to the extensive studies on mammalian/human counterparts, comprehensive understanding on plant CB5 functions has not been achieved so far. Sporadic evidence reveal that plant CB5s robustly interact with different types of proteins ranging from catalytic enzymes such as P450 monooxygenases, desaturases, reductases, hydroxylases, to transporters, stress response – or hormone signaling regulators. Such versatile but most likely transient physical interactions infer that plant CB5s not only simply act as electron carriers modulating enzymatic catalyses and metabolic processes but may also serve as the cellular regulators or modulators that connect and coordinate different biological processes in response to the environmental stresses, and/or to the fluctuation of cellular redox and carbon status. A summary of plant CB5 functions are depicted in Figure 2. Nevertheless, to date we still lack a comprehensive insight into the biochemical and biological significance of plant CB5 proteins. Many open questions remain to be addressed to deeply understanding CB5 functionalities in plants. For instance, (1) With multiple CB5 family members that plants evolved, do they play distinct biological functions or act redundantly in plant metabolisms? (2) As redox factors, what are the additional metabolic and cellular processes that CB5 proteins are involved in? (3) As an electron shuttle component, do the CB5 proteins associate with different electron transport pathways for different classes of metabolic processes? (4) What is the biochemical significance of a P450 system that necessarily recruits CB5 protein in an electron transport pathway instead employs the typical NADPH-CPR electron transfer chain? (5) What is the molecular and structural basis for a P450 system necessitating CB5 proteins? The insight gained from the exploration of this family of heme-containing proteins might offer more sophisticated and versatile molecular tools to augment our ability in engineering plant metabolism to effectively utilize the photosynthetically fixed reduced carbon and reducing power for the production of desired bioproducts.

## Author contributions

The author confirms being the sole contributor of this work and has approved it for publication.
